# Tips for pandemic response planning for Internal Medicine training programs

**DOI:** 10.15694/mep.2020.000182.1

**Published:** 2020-08-27

**Authors:** Carleen Spitzer, Jennifer Allen, Tejas Sinha, Devin Haddad, Ellen Liu, David Wininger, Lisa Kearns, Jared Moore, Allison Rossetti

**Affiliations:** 1 The Ohio State University Wexner Medical Center

**Keywords:** Medical education research, planning, Internal medicine, pandemic, COVID-19

## Abstract

This article was migrated. The article was marked as recommended.

The current Coronavirus Disease 2019 (COVID-19) pandemic has strained hospital systems and training programs across the world. As capacity issues mount and trainees are called upon to provide frontline medical care, programs and institutions have had to rapidly evolve to redefine the trainee experience. To that end, there is a paucity of literature regarding how healthcare training programs should operate during a global pandemic. Here, we aim to describe twelve evidence-based recommendations for coordinating a cohesive, systematic approach to pandemic response planning for Internal Medicine residency training programs. These tips encompass inpatient and outpatient practices, provider safety, resuscitation, virtual education programming and resident wellbeing. Though many of these considerations or recommendations were not described during the COVID-19 pandemic, these tips have been described previously in the literature, are applicable to the current pandemic and could be easily extrapolated to future crises.

## Introduction

On March 11, 2020, the World Health Organization declared Coronavirus Disease 2019 (COVID-19) a global pandemic. As of May 27, there have been 5,488,825 cases of COVID-19 with 349,095 deaths, and the numbers are still multiplying ( World Health Organization, 2020). The COVID-19 pandemic has caused disruptions in every facet of daily life with residency training programs being no exception. With their trainees constituting a large portion of frontline providers, Internal Medicine training programs have required rapid evolution in how they carry out their mission of preparing the next generation of internists.

There is little available data to advise healthcare training programs on best practices and procedures during this global pandemic. Though some literature exists regarding training program operations during the severe acute respiratory syndrome (SARS) and Middle Eastern respiratory syndrome (MERS) outbreaks ( Sherbino and Atzema, 2004), these epidemics pale in comparison to the current pandemic that the world is experiencing ( Chan-Yeung and Xu, 2003; World Health Organization, 2017).

Over the past several weeks, more and more is being written about the impact of the virus on various training programs and their trainees ( Balakrishnan et al., 2020; Cleland et al., 2020; Goel and Sharma, 2020). Despite this, there is still a lack of literature regarding how programs may identify various challenges and then combat them. Here, we describe 12 tips designed specifically for Internal Medicine programs to provide them with a framework as they address the challenges and opportunities involved in pandemic planning. Though many of these considerations or recommendations were not described during the COVID-19 pandemic, these tips have been described previously in the literature, are applicable to the current pandemic and could be easily extrapolated to future crises.

### Tip 1

#### Share and coordinate residency leadership responsibilities

During pandemic planning, program leadership needs to make effective use of existing team members including program directors, chief residents, program administrators and key faculty while extending expertise by tapping into new and existing contacts within and outside the local institution. The program director should not take primary responsibility for all decisions or tasks. Increased delegation to content experts concerning resident schedules, changes to inpatient and outpatient services, adjustments to structured educational offerings, instructions and monitoring of safety practices and policies and best practices for clinical care should be considered. A shared approach distributes the time burden involved to address the multiple facets involved in crisis planning ( Aufegger et al., 2019; Bienefeld and Grote, 2013; Deitchman, 2013; Wang, Waldman and Zhang, 2013). Involving multiple parties offers a diverse approach to problem analysis and affords increased creativity in developing effective solutions. It remains essential that the program director and others on the leadership team are aware of the direction of change in the various domains and overall institutional master plans. Frequent communication between involved parties via regular email or virtual meetings allows stakeholders to provide input on policies and evaluate possible unintended effects in other critical domains.

### Tip 2

#### Ensure residency program representation during medical center preparedness planning

In response to a pandemic, hospitals must strategize and implement a preparedness plan focused on patient triage, care capacity and provision of appropriate staffing during a potential patient surge. Fundamental to this effort is the formulation of an operations task force including key frontline personnel ( Chopra et al., 2020). Internal Medicine residents are probable frontline workers and have the potential to comprise a large percentage of the inpatient work force. As such, residency program representation and involvement in the preparedness planning is essential.

When involved in hospital preparedness working groups, Internal Medicine residency programs have the opportunity to collaborate with hospital leadership to ensure that preparedness plans abide by regulations imposed by training oversight committees and meet the common goal of providing safe and effective patient care. Programs need to be aware of which key stakeholders anticipate having Internal Medicine resident support in the event of a patient surge to effectively operationalize resident deployment. Attention should be paid to trainee safety and supervision, appropriate provider to patient ratios and adherence to imposed regulations. Lastly, programs should reflect on their core values and ensure that these specific values are also supported.

### Tip 3

#### Adjust resident schedules to limit pandemic exposure and develop a comprehensive back up system

There is not yet consensus surrounding the extent to which resident and fellow trainees should be shielded from participating in the care of patients infected with COVID-19. In the United States, the Accreditation Council for Graduate Medical Education (ACGME) supports trainee involvement in the care of COVID-positive patients if they receive adequate training in infection control and disease management, comply with established work hour restrictions and receive direct supervision from faculty ( ACGME, 2020). Depending on local need, health systems may choose to divert trainee involvement for purposes of both protecting trainees from pandemic exposure as well as limiting personal protective equipment (PPE) utilization.

With the potential for a steep influx of ill patients, it is critical to be able to expand inpatient service lines with a particular focus on critical care units to meet evolving health system demands. In a prevailing pandemic care model, as the patient surge intensifies, additional providers with appropriate training should be deployed in a tiered fashion to increase service line capacity ( Augoustides, 2020). Residency programs may consider removing residents from non-essential rotations such as consult services and clinic electives to expand their available healthy provider pool. Residents removed from scheduled rotations should remain active with structured educational plans, telehealth visits and professional development. Additionally, programs should develop clear policies and communication regarding trainees who have conditions making them more vulnerable to complications from COVID-19. A range of accommodations can be considered based on the individual’s risk factors and preferences including removal from all direct patient care duties or assignment to services that are free of COVID-positive patients.

### Tip 4

#### Educate trainees on proper handwashing as well as donning and doffing technique

Regardless of a health system’s anticipated trainee involvement in the care of patients infected with COVID-19, appropriate education on hand hygiene and utilization of PPE is of upmost importance. Incorrect use of PPE increases trainee exposure risk and could contribute to COVID-19 transmission. Despite its critical importance in protecting healthcare providers and patients, studies suggest that errors in donning and doffing PPE occur frequently ( Tomas et al., 2015; Zellmer, Hoof and Safdar, 2015). In a simulation designed to enhance infection control processes within the intensive care unit, participants were noted to make a number of errors related to donning and doffing PPE. Noted errors included incorrectly removing contaminated gloves, cross-contamination due to team member proximity and failure to identify the correct sequence of donning and doffing PPE ( Choi et al., 2020). This study highlights the necessity of reviewing donning and doffing procedures with all trainees to eliminate common errors and improve healthcare worker safety. Training can be accomplished in a myriad of ways including direct observation with feedback, simulation or via lecture format or training video followed by written assessment to verify competency.

### Tip 5

#### Adapt resuscitation policies to fit the changing environment

Recent estimates suggest that the mortality rate of COVID-19 is approximately 3% ( Rajgor et al., 2020) and mortality rates following in-hospital cardiac arrest are expected to be higher than usual in-hospital mortality given the severity of illness of these patients and expected delays in initiating resuscitation due to necessary donning of PPE ( Chan, Berg and Nadkarni, 2020). Though there is some debate regarding the extent to which chest compressions and defibrillation should be considered aerosolizing procedures ( Couper et al., 2020; Iacobucci, 2020), there is consensus that members of resuscitations teams are at increased risk of contracting COVID-19 for a multitude of reasons ( Lockhart et al., 2020; Edelson et al., 2020). Given the concern for infection transmission, current institutional resuscitation practices should be examined to ensure that the immediate needs of patients are balanced with the safety of healthcare providers. In general, when providing resuscitative care for patients who are known or suspected to be COVID-19 positive, a concerted effort should be made to limit the number of providers involved and to ensure that all providers have appropriate PPE donned prior to entering the patient care area. Oxygenation and ventilation modalities associated with lower aerosolization risk should be implemented, and the risks versus benefits of initiating and continuing resuscitation efforts given each patient’s clinical condition should be continually reexamined ( Edelson et al., 2020). Additionally, trainees themselves have advocated for an increased focus on goals of care discussions either within the outpatient setting or when patients initially present as a means to decrease the frequency of resuscitative events - especially if these occurrences are not in line with patients’ goals ( DeFilippis, Ranard and Berg, 2020).

### Tip 6

#### Create rounding principles for teaching services

Inpatient rounds are the cornerstone of inpatient medical care and medical education in teaching hospitals. Medical educators have advocated for attending rounds to be conducted at the bedside to enhance teaching, allow for learner assessment, improve communication and optimize patient-centered care ( Carlos et al., 2016; Lichstein and Atkinson, 2018). With social distancing guidelines and efforts to conserve and extend PPE, the vision of the large multidisciplinary team shoulder to shoulder at the patient’s bedside are no longer the ideal ( CDC, 2020). Recognizing that traditional rounding styles may not align with new values and priorities, Internal Medicine residency programs should develop new rounding guidelines to assist faculty and trainees as they navigate changes to prior practice. Programs should be specific in outlining rounding style options that would satisfy the core tenets of patient care while heightening provider and patient safety and preserving hospital resources. Guidelines should provide suggestions to maintain engagement in the educational process as well as strategies and resources to mitigate the negative unintended consequences of altering rounding routines. Novel approaches such as utilization of inpatient video visits to enhance communication beyond the bedside evaluation and best practices for communication with patients and families should be included. Creating a shared mental model for best rounding practices during the COVID-19 pandemic avoids trainee uncertainty, creates predictability across the health system and allows for best practice refinement through feedback of shared experience.

### Tip 7

#### Standardize socially distanced sign-outs

Miscommunications during patient handoffs between trainees is a leading cause of medical errors. The ACGME Clinical Learning Environment Review outlines the need for education in care transitions and standardized handoffs in resident education ( ACGME, 2018). Studies support the utilization of standardized handoff training and formats as these interventions have been shown to increase trainee comfort and decrease medical errors ( Lescinskas, Stewart and Shah, 2018; Starmer et al., 2014).

In the era of COVID-19, the need for social distancing has provided an additional challenge when it comes to standardized care transitions between trainees who often work in confined spaces and gather in groups during handoff. It is imperative to establish a protocol for effective and safe patient care transition while protecting trainees from unnecessary exposure. Trainees can be encouraged to adhere to social distancing guidelines by conducting handoffs via phone or video communication, maintaining updated written sign-outs in the electronic medical record for cross-covering teams to reference and communicating via standardized formats to ensure safe transitions of care.

### Tip 8

#### Adjust clinic schedules to preserve resident workforce while maximizing teamwork and patient safety

As significant adjustments are made to inpatient operations, training programs should also adapt their ambulatory care practices and curriculum to meet the needs of patients, trainees, clinic staff and faculty preceptors. Clinic schedules should be modified to decrease the burden of in-person visits in an effort to minimize COVID-19 exposure risk while continuing to provide optimal patient care. Rescheduling non-urgent appointments and converting appointments to telehealth or virtual visits as clinically appropriate allows for a decrease in onsite staffing while allowing for primary care access ( Ambert-Pompey et al., 2017). Truncated in-person schedules free residents working in their primary clinic to provide paperwork cross coverage for colleagues assigned to work remotely. Residents assigned to specialty clinics in which they are not the primary provider may still be able to participate in virtual visits or in an educational plan that would meet the core objectives of the rotation. Those previously scheduled to work in clinic may be redeployed to manage the electronic inbox, conduct virtual visits remotely or be utilized in the inpatient arena.

### Tip 9

#### Utilize telemedicine to optimize the primary care experience during social distancing

Internationally the use of telehealth has rapidly increased to meet the unique challenges posed by the COVID-19 pandemic ( Ohannessian, Duong and Odone, 2020). Healthcare systems that have not previously utilized telehealth are faced with the challenge of adopting new technology as regulating bodies approve its use and reimbursement ( Rockwell and Gilroy, 2020). In an effort to minimize disruptions in training and continue primary care access to the community, residents should be incorporated into the telehealth workflow ( Oldenburg and Marsch, 2020). Depending on the modality, preceptors may be able to supervise multiple trainees simultaneously. Best practices, including aspects such as choosing a care delivery platform and understanding government regulations, should be created with a focus on providing appropriate supervision while optimizing the patient experience. Incorporating such foundational basics of telemedicine in the core ambulatory clinic curriculum may also serve as another modality for teaching systems-based practice and healthcare disparities when usual methods may not be possible during social distancing.

### Tip 10

#### Provide diverse easily implemented virtual learning opportunities

The COVID-19 pandemic has forced residency programs nationwide to suspend in-person educational activities and rapidly innovate virtual education curricula. As resident wellness and sense of community is negatively impacted by the disruption of conventional face-to-face conferences, programs should aim to transition their regular interactive didactics to interactive web-based platforms ( Bolster and Rourke, 2015). Core liaisons or faculty leads should be responsible for collating asynchronous learning materials to promote mastery of intended rotation objectives for trainees assigned to distance learning. Virtual education can be augmented by utilizing popular Free Open Access Med (FOAM) podcasts and websites, as most trainees are comfortable with these platforms and able to utilize them easily ( Sterling et al., 2017). Programs can consider using the “Flipped Classroom” model in conjunction with these resources to maximize their educational value as it has been shown to improve learning in health professions education( Hew and Lo, 2018). To easily implement this model, educators can create 5-10 “Flipped Classroom” style multiple-choice questions for each resource and disseminate them via twitter polls, email or internal program webpages. This system allows trainees to learn at their own pace - minimizing cognitive overload while providing frequent testing and making use of the retrieval practice effect( Bolster and Rourke, 2015).

### Tip 11

#### Support residents during a pandemic

By virtue of their role within the health system, residents are at high risk for developing burnout during periods of routine operation ( IsHak et al., 2009) let alone during periods of heightened anxiety and stress. Programs should develop systems to attend to residents’ emotional and physical wellbeing in conjunction with their efforts to preserve their educational experience during the pandemic. They should consider ways to optimize and streamline communication including opportunities for trainees to interact with program leadership. Virtual office hours can be used to maintain social connection despite physical distance, address queries and concerns and assess recent program changes to allow for quick adaptation. Establishing trainee “battle buddies” or small trainee units charged with peer-to-peer support can provide an additional layer of accountability and aide program directors in identifying residents who may benefit from additional intervention ( Hertling, 2020). To mitigate ill effects of physical and emotional fatigue, programs may choose to adjust schedules to limit consecutive workdays, shorten shift duration or provide other creative solutions to ensure residents are receiving adequate respite during periods of increased stress. Systems should facilitate easy access to mental health services to address psychological distress and routine structured debriefing sessions should be in place ( Costa and Moss, 2018; Knobler et al., 2007). Lastly, programs should have a clear plan for when trainees warrant viral testing as well as a protocol for accidental exposures and acquired illness as even the fear of potential exposure or illness can be anxiety-producing and can negatively affect residents’ mental wellbeing.

Knobler HY, Nachshoni T, Jaffe E, Peretz G, Yehuda YB. Psychological guidelines for a medical team debriefing after a stressful event. Mil Med. 2007;172(6):581-5.Knobler HY, Nachshoni T, Jaffe E, Peretz G, Yehuda YB. Psychological guidelines for a medical team debriefing after a stressful event. Mil Med. 2007;172(6):581-5.Knobler HY, Nachshoni T, Jaffe E, Peretz G, Yehuda YB. Psychological guidelines for a medical team debriefing after a stressful event. Mil Med. 2007;172(6):581-5.Knobler HY, Nachshoni T, Jaffe E, Peretz G, Yehuda YB. Psychological guidelines for a medical team debriefing after a stressful event. Mil Med. 2007;172(6):581-5.Knobler HY, Nachshoni T, Jaffe E, Peretz G, Yehuda YB. Psychological guidelines for a medical team debriefing after a stressful event. Mil Med. 2007;172(6):581-5.

### Tip 12

#### Prepare for the future: our new normal

As the first wave of the COVID-19 pandemic subsides and plans for the next academic cycle begin, Internal Medicine residency program leadership should reevaluate their initial pandemic planning response. Ongoing adjustments with the potential of additional waves of disease or outbreaks should be considered with attention to the need for restructuring as new trainees who would require more oversight and support matriculate. Onboarding of new trainees may require virtual elements to avoid large group gatherings and include more rigorous training in proper PPE use ( Tomas et al., 2015). Programs need to develop new best practices to facilitate recruitment in a virtual era that is fair and equitable to students and programs alike ( AAMC, 2016, 2020). In the same vein, they will need to assist senior trainees as they navigate the fellowship and job application processes ( Jones and Abdelfattah, 2020). Finally, programs will have to evaluate which new initiatives such as telehealth and novel education efforts might merit continued use given their newly demonstrated value.

## Conclusion

COVID-19 has severely disrupted healthcare delivery systems around the world, and the impact of this disease remains far-reaching. In order to address the current pandemic and potential future crises, training programs need to be thoughtful about the effects on both their learners and the clinical environment in which their trainees practice. These 12 recommendations provide a framework for program directors as they consider the many facets of their training program that may be affected by a pandemic as well as ways in which current practices may be adapted. These tips are not exhaustive but are meant to serve as a basis for initial evaluation and programmatic improvement.

## Take Home Messages

• Training programs should develop an evidence-based, systematic approach as they consider how their particular program will need to adapt to a pandemic or similar crisis
• Points to consider include inpatient and outpatient practices, provider safety, resuscitation practices, virtual education programming and resident wellbeing
• It is important that programs reexamine their initial pandemic response and adjust practices as indicated
• As we cross over into the new academic year during the current pandemic, programs should be developing plans to transition new trainees into the work force and devising plans how best to prepare current residents for the next phase in their careers

## Notes On Contributors

Carleen Spitzer, MD is an Assistant Professor in the Division of Pulmonary and Critical Care Medicine and an assistant program director of the Internal Medicine Residency Program at The Ohio State University Wexner Medical Center.

Jennifer Allen, MD is an Assistant Professor in the Division of Hospital Medicine and an associate program director of the Internal Medicine Residency Program at The Ohio State University Wexner Medical Center.

Tejas Sinha, MD is one of the chief internal medicine residents at The Ohio State University Wexner Medical Center.

Devin Haddad, MD one of the chief internal medicine residents at The Ohio State University Wexner Medical Center.

Ellen Liu, MD is one of the chief internal medicine residents at The Ohio State University Wexner Medical Center.

David Wininger, MD is the Internal Medicine Residency program director and an Associate Professor in the Division of Infectious Diseases, Department of Internal Medicine at the Ohio State University Wexner Medical Center.

Lisa Kearns, MD, MS is an Assistant Professor in the Division of General Internal Medicine, program director for the Primary Care Track Program and associate program director of the Internal Medicine Residency Program at The Ohio State University Wexner Medical Center.

Jared Moore, MD is an Associate Professor in the Division of General Internal Medicine and an associate program director for the Internal Medicine Residency Program.

Allison Rossetti, MD is an Assistant Professor in the Divisions of Adult and Pediatric Hospital Medicine and assistant program director of the Internal Medicine and Pediatric Residency Program at The Ohio State University Wexner Medical Center and Nationwide Children’s Hospital.
