# Supplementary material for: Tips for pandemic response planning for Internal Medicine training programs
Source: MedEdPublish (2016). 2020 Aug 27;9:182. [Version 1] doi: 10.15694/mep.2020.000182.1 (PMC10702634; doi:10.15694/mep.2020.000182.1)
Supplement: Peer review history

# Reviewer report: Reviewer response for version 1

**DOI:** 10.21956/mep.20114.r31021

**Published:** 2020-10-07

**Authors:** Trevor Gibbs

**Affiliations:** 1 AMEE

This review has been migrated. The reviewer awarded 4 stars out of 5 An interesting paper that joins the large number of other papers related to management of individuals and groups during the Covid-19pandemic. The group chosen here are internal medicine residents, a group not frequently spoken of. The tips represent a logical approach to care and safety of the residents and although a little US-centric, they are transferable to other forms of junior staff.I would hope now that these authors begin to evaluate their actions to see if these tips really are of value and also how they have implemented them.I would recommend this paper to all involved in resident training, particularly during this very difficult time

Reviewer Expertise:

NA

No decision status is available

# Reviewer report: Reviewer response for version 1

**DOI:** 10.21956/mep.20114.r31019

**Published:** 2020-09-06

**Authors:** John Cookson

**Affiliations:** 1 University of Worcester

This review has been migrated. The reviewer awarded 4 stars out of 5 Thank you; there are many useful ideas here about continuing to manage the experience of junior staff so that patient care is continued, education is preserved and everyone is kept as safe as possible. Although there is now an extensive literature on changes to UG teaching, especially assessment, there has been less on managing the inevitable conflicts which arise in situations where care givers are also trainees.

Reviewer Expertise:

NA

No decision status is available

# Reviewer report: Reviewer response for version 1

**DOI:** 10.21956/mep.20114.r31020

**Published:** 2020-09-03

**Authors:** Wai Ching Lee

**Affiliations:** 1 Sengkang General Hospital

This review has been migrated. The reviewer awarded 4 stars out of 5 Thank you for the article. There are several useful tips highlighted in this article especially in terms of well being of the residents. Moving forward perhaps just 2 pointers to possibly elaborate further 1) Possible suggestions to mitigate the issue of patient consent/ privacy to be part of telemedicine / e- learning 2) Quality of residents who graduate with these limitations during pandemic era towards becoming a specialist in the future.

Reviewer Expertise:

NA

No decision status is available
